# Effect of Calisthenics on Sleep Quality and Well-Being Among Older Adults

**DOI:** 10.7759/cureus.76821

**Published:** 2025-01-02

**Authors:** Vishal K Ghorpade, Shilpa P Satralkar, Shardul Jadhav

**Affiliations:** 1 Medical Surgical Nursing, Bharati Vidyapeeth (Deemed to be University) College of Nursing, Sangli, IND

**Keywords:** calisthenics, effect, older adults, quality of sleep, well-being

## Abstract

Introduction: This is a study to assess the effect of selected calisthenics on the quality of sleep and well-being among old-age individuals in the selected old-age home at Sangli, Miraj, Kupwad Corporation area. The objective is to assess the quality of sleep and well-being in the experimental and control groups before the intervention and the level of sleep and well-being in the experimental and control groups after the intervention.

Methodology: The study used a quasi-experimental one-group pre-test post-test design. Using a simple random sampling technique, 60 participants were selected. Calisthenics exercises were implemented to improve sleep quality and well-being. On the eighth day after the calisthenics program, a post-test was conducted. The analysis was performed using frequency and percentage distribution and a paired t-test.

Results: Post-intervention, the Pittsburgh sleep quality index (PSQI) score and quality of sleep among old-age individuals in the experimental group were 12 (40%) had poor sleep and 18 (60%) had a good quality of sleep among old-age people. The mean was 4.26, S.D. was 1.7207, and in the control group, the mean was 8.23, S.D. was 2.4166, and the t-value was 7.3234. P-value was 0.00001 < 0.05 (at 5% level of significance). The well-being score among old-age individuals in the experimental group after the intervention was that there were three (10%) who had very good well-being, 24 (80%) had good well-being scores, and three (10%) had poor well-being among elderly people. The mean was 129, S.D. was 13.3209, and in the control group, the mean was 110.3, S.D. was 8.065, the t-value was -6.5773, and the p-value was 0.00001 < 0.05 (at a 5% level of significance). This reveals that the effect of calisthenics on the quality of sleep and well-being among old age individuals was effective.

Conclusion: The study concluded that planned calisthenics exercises were implemented to enhance sleep quality and well-being. Elderly individuals responded positively to the program, showing improvements in both sleep quality and well-being after participating in the exercises.

## Introduction

The physical, psychological, and even economic and environmental changes that emerge with age cause problems in sleep. The lack of exercise habits could increase the incidence of unfavorable sleep disorders with depression and fatigue [1].

Old age is the final stage of life and is considered a despised period. The world's population of 60 years and over is expected to increase from 605 million to two billion in 2050 (WHO) [2,3]. Population Reference Bureau predicts an increase in the Indian elderly population from 8% in 2010 to 19% in 2050 [4].

Aging is a continuous process that begins at conception and ends with death. Several factors influence the development of self-esteem; among them are the respect, acceptance, and attention received from significant individuals in one’s life, including family members [5].

However, few studies have included the oldest-old, above 85 years old, although older persons span a wide range of life years. Older adults will experience physical, psychological, social, and spiritual effects from aging. Physically older people become less active as they age [6,7].

Aging-related changes in the body typically increase a person's susceptibility to a wide range of illnesses as well as the negative consequences and difficulties of medical care. Their response times become slower with age, thus it could take them longer to adapt to changes in their surroundings [8]. The most prevalent conditions affecting the elderly are hearing loss (8.2%), neurological complaints (18.7%), visual impairment (88%), locomotor disorder (44%), and respiratory disorders (16.1%) [9].

To enhance elderly individuals' feelings of well-being through physical activity and better sleep, therefore, the researcher desired to take action for this population's benefit. Thus, the investigator was compelled to evaluate the impact of particular exercises aimed at enhancing the well-being and sleep quality of senior citizens residing in assisted living facilities.

## Materials and methods

This study employed a quasi-experimental one-group pre-test post-test design to evaluate the effect of selected calisthenics exercises on sleep quality and well-being among elderly individuals. The research was conducted in the Sangli, Miraj, and Kupwad corporation areas, focusing on the specific needs of this population. The study targeted elderly individuals aged 60 to 75 years, including both male and female participants, and a total of 60 individuals were recruited using a simple random sampling technique to ensure an unbiased representation of the target population. To determine the appropriate sample size, a power analysis was conducted, resulting in 30 participants allocated to the experimental group and 30 to the control group. Elderly participants aged 60 to 75 years who demonstrated a willingness to participate and were physically and mentally capable of performing the prescribed exercises were included in the study. Bedridden individuals, unable to sit unassisted, mentally incompetent, or unable to perform exercises or follow commands were excluded from participation.

The participants' baseline sleep quality and well-being were assessed using standardized instruments during the assessment phase. The intervention involved a structured calisthenics exercise program tailored to the physical capabilities of older adults, which was implemented regularly, with an assessment conducted on the eighth day to evaluate the outcomes focusing on the impact of the intervention on sleep quality and well-being. The calisthenics exercise program included simple, low-impact exercises designed to enhance sleep quality and well-being while prioritizing safety, with each session supervised to ensure adherence to the protocol and minimize the risk of injury.

The institutional ethics committee of Bharati Vidyapeeth (Deemed to be University), College of Nursing, Sangli, approved the study (IECBVDUCON, Sangli Maharashtra Reg. No. EC/NEW/INST/2024/MH/0414), and informed consent was obtained from all participants, who were thoroughly informed about the study's objectives, procedures, and their right to withdraw at any time without penalty. Data were collected, the study was summarized using frequency and percentage distribution, and a paired t-test was conducted to determine the statistical significance of changes in sleep quality and well-being between the pre-test and post-test assessments, with statistical significance set at p < 0.05.

## Results

The results section is organized into various categories. It begins with the frequency and percentage distribution of demographic variables. The assessment of sleep quality follows, focusing on evaluating the levels of sleep quality in the experimental and control groups both before and after the intervention. Next, the assessment of well-being is addressed, examining the levels of well-being in the experimental and control groups before the intervention and again after the intervention. Finally, the section concludes with a comparative analysis, highlighting differences in the quality of sleep and well-being between the experimental and control groups after the intervention. 

Among the total sample, in the experimental group, 7% of the sample belongs to the age group between 60 and 65 years, 43% between 66 and 70 years, 33% between 71 and 75 years, and 20% between 76 and 80. Whereas in the control group, 23% were between 60 and 65 years, 27% between 66 and 70 years, 43% between 71 and 75 years, and 76 and 80 years with 7%. Regarding the gender of the old age individuals, in the experimental group, 43% of the samples are male and 57% are female, whereas in the control group, 53% are male and 47% are female. Regarding engaging in physical activity in old age, individuals in the experimental group: 63% of samples are engaging in physical activity, and 37% are not engaging in physical activity. Whereas the control group has 67% of samples engaging in physical activity and 33% not engaging in physical activity. Regarding staying in old age homes in the experimental group, 20% of samples stayed less than one year, 40% were one to three years, and 40% were above three years. Whereas in the control group, 60% of samples are staying less than one year, 23% are one to three years, and 17% are staying above three years. The details of the pre-test assessment distribution of demographical variables are summarized in Table 1.

**Table 1:** Frequency and percentage distribution of demographical variables

Demographical variables		Experimental group		Control group	
		Frequency	%	Frequency	%
Age (in years)	60–65	2	7	7	23
	66–70	13	43	8	27
	71–75	9	30	13	43
	76–80	6	20	2	7
Gender	13	43	16	53	13
	17	57	14	47	17
Regular exercises	Yes	19	63	20	67
	No	11	37	10	33
	Less than 1 yr.	6	20	18	60
Staying in the old age home (in yrs.)	1–3 yrs.	12	40	7	23
	Above 3 yrs.	12	40	5	17

**Figure 1:**
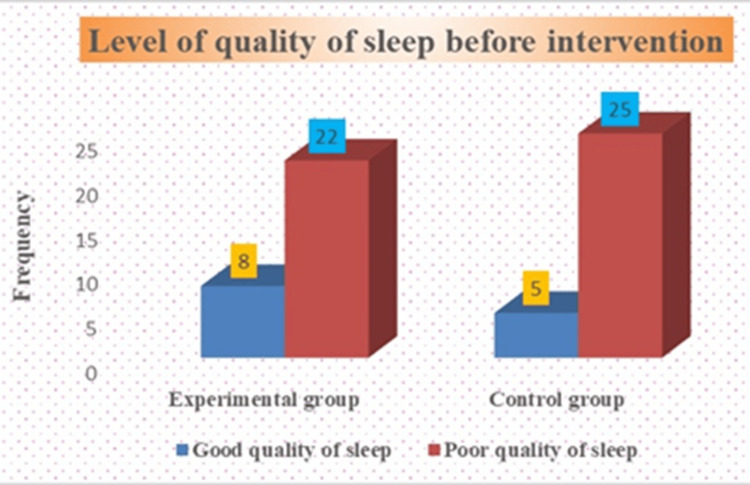
Assessment of the level of quality of sleep in experimental and control groups before intervention

According to the PSQI score in the experimental group, most of the 22 (73%) old-age individuals had poor quality of sleep, and eight (27%) had good quality sleep before intervention. In the control group, most of the 25 (83%) had poor quality sleep, and five (17%) had good quality sleep before the intervention among old people. These baseline findings of sleep quality are presented in Figure 1.

**Figure 2:**
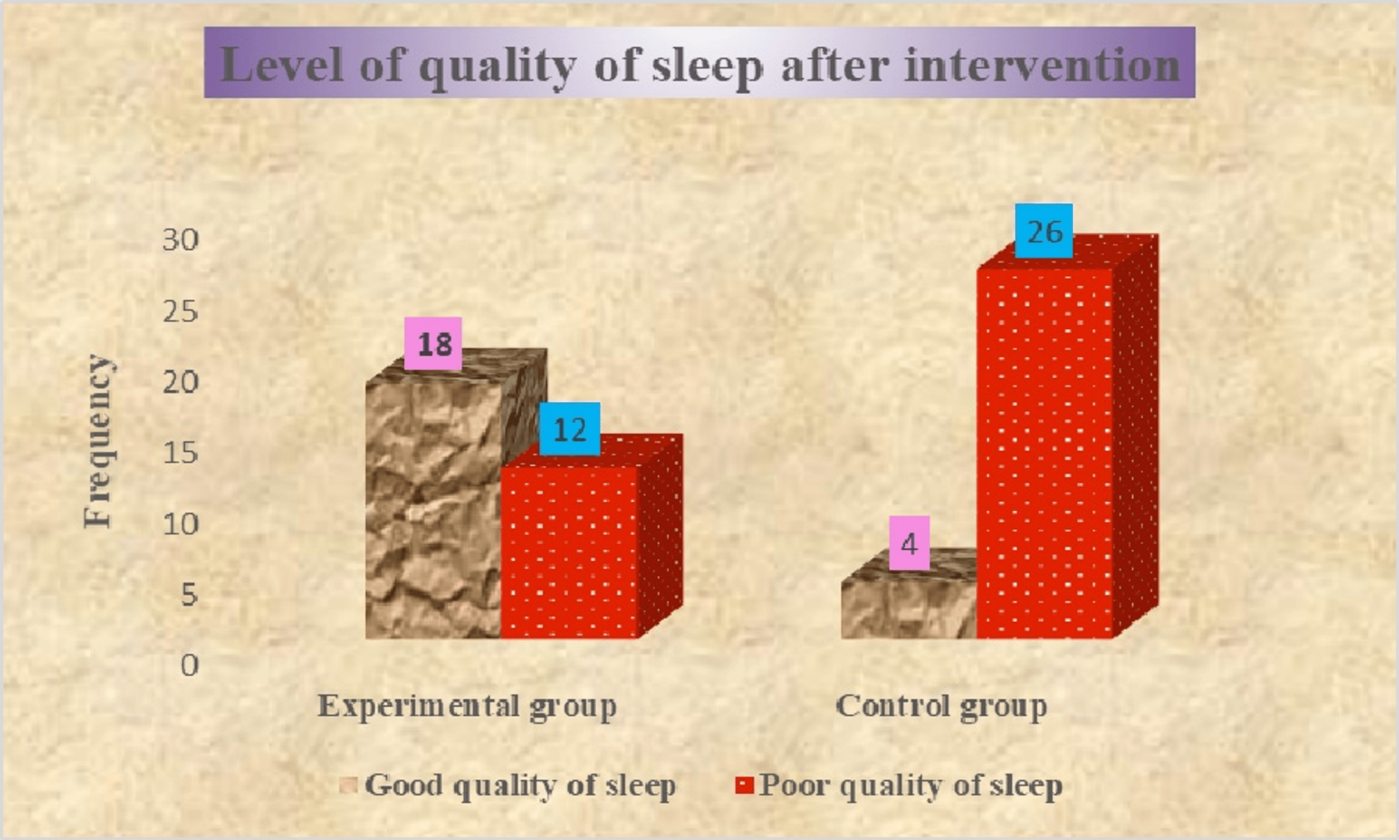
Assessment of the level of quality of sleep in experimental and control groups after intervention

According to the PSQI score in the experimental group after calisthenics exercise, there were 12 (40%) who had poor sleep quality and 18 (60%) who had a good quality of sleep among old age people. According to the PSQI score, most of the old-age individuals, 26 (87%), had poor and four (13%) had good quality sleep in the control group among old-age people. The outcome findings of sleep quality after the intervention are presented in Figure 2.

**Figure 3:**
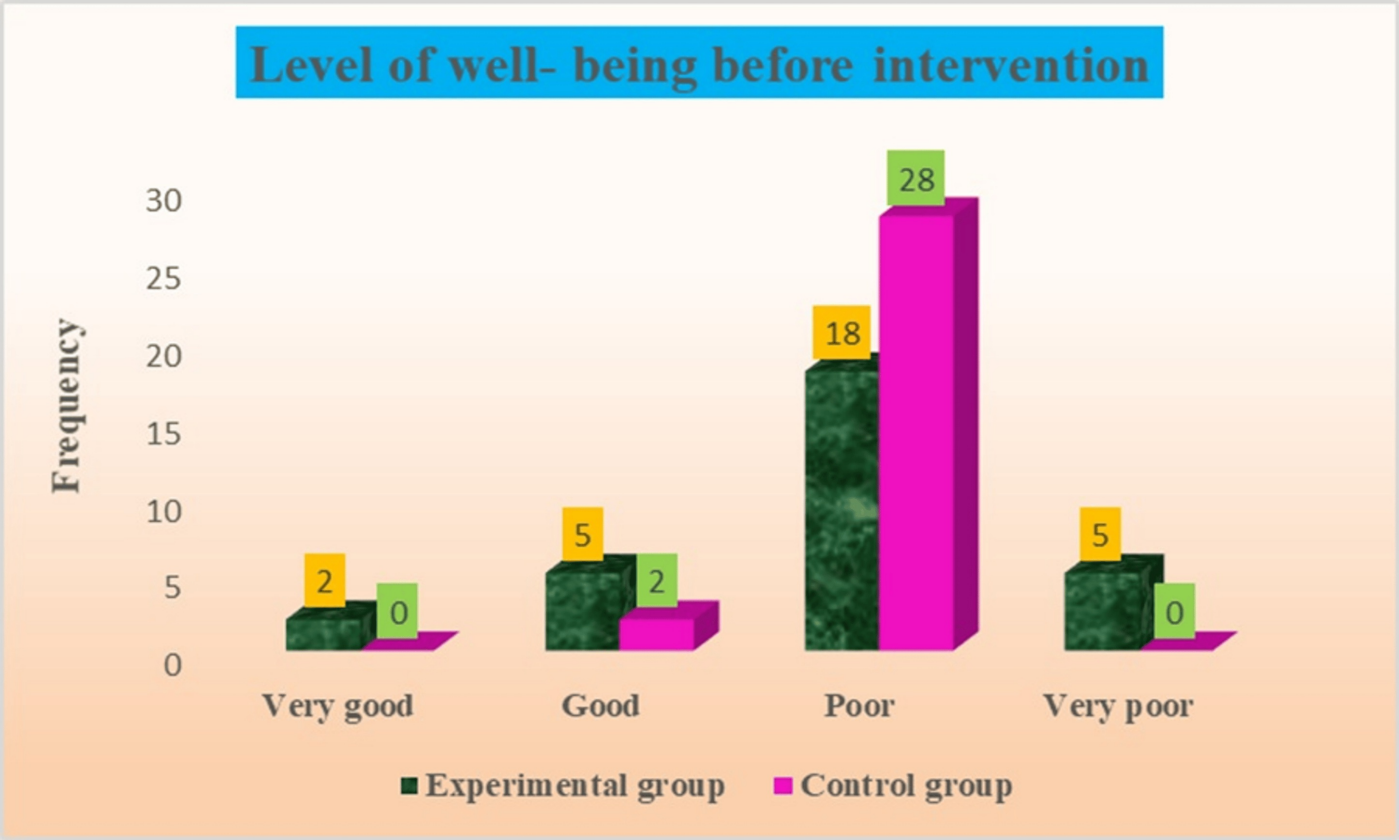
Assessment of the level of well-being in experimental and control groups before intervention

According to the well-being scale in the experimental group before the intervention, there were 18 (60%) who had poor well-being, five (7%) who had very poor and good well-being, and two (7%) who had very good well-being among old age people. According to the well-being scale, 28 (93%) old-age individuals had poor well-being, and two (7%) had good well-being in the control group before the intervention among old-age people. These baseline findings of well-being are presented in Figure 3.

**Figure 4:**
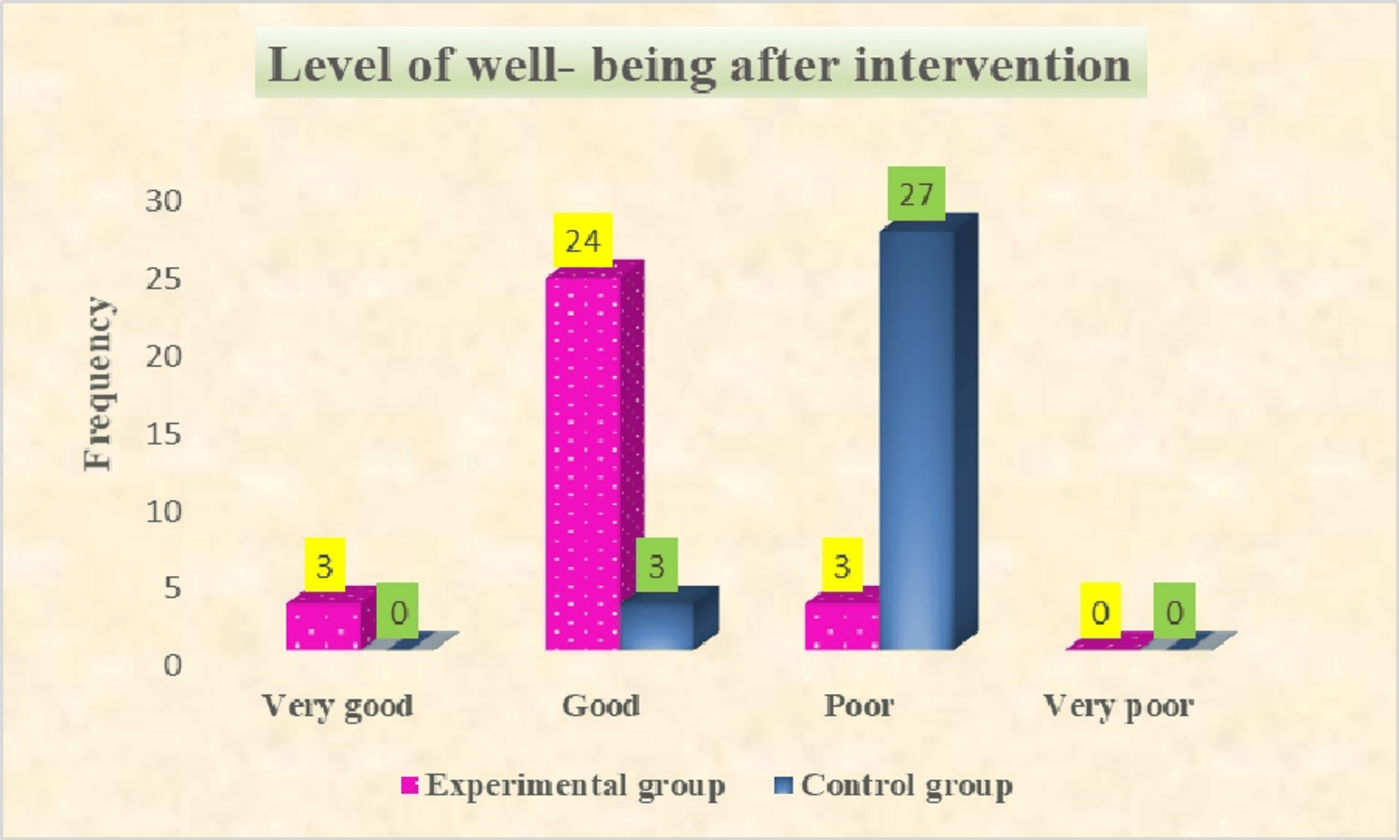
Assessment of the level of well-being in experimental and control groups after intervention

According to the well-being scale in the experimental group after the intervention, there were three (10%) who had very good and poor well-being, and 24 (80%) had good well-being scores among old-age people. According to the well-being scale, most of the 27 (90%) old-age individuals had poor well-being in the post-test of the control group. The outcome findings of well-being after the intervention are presented in Figure 4.

**Figure 5:**
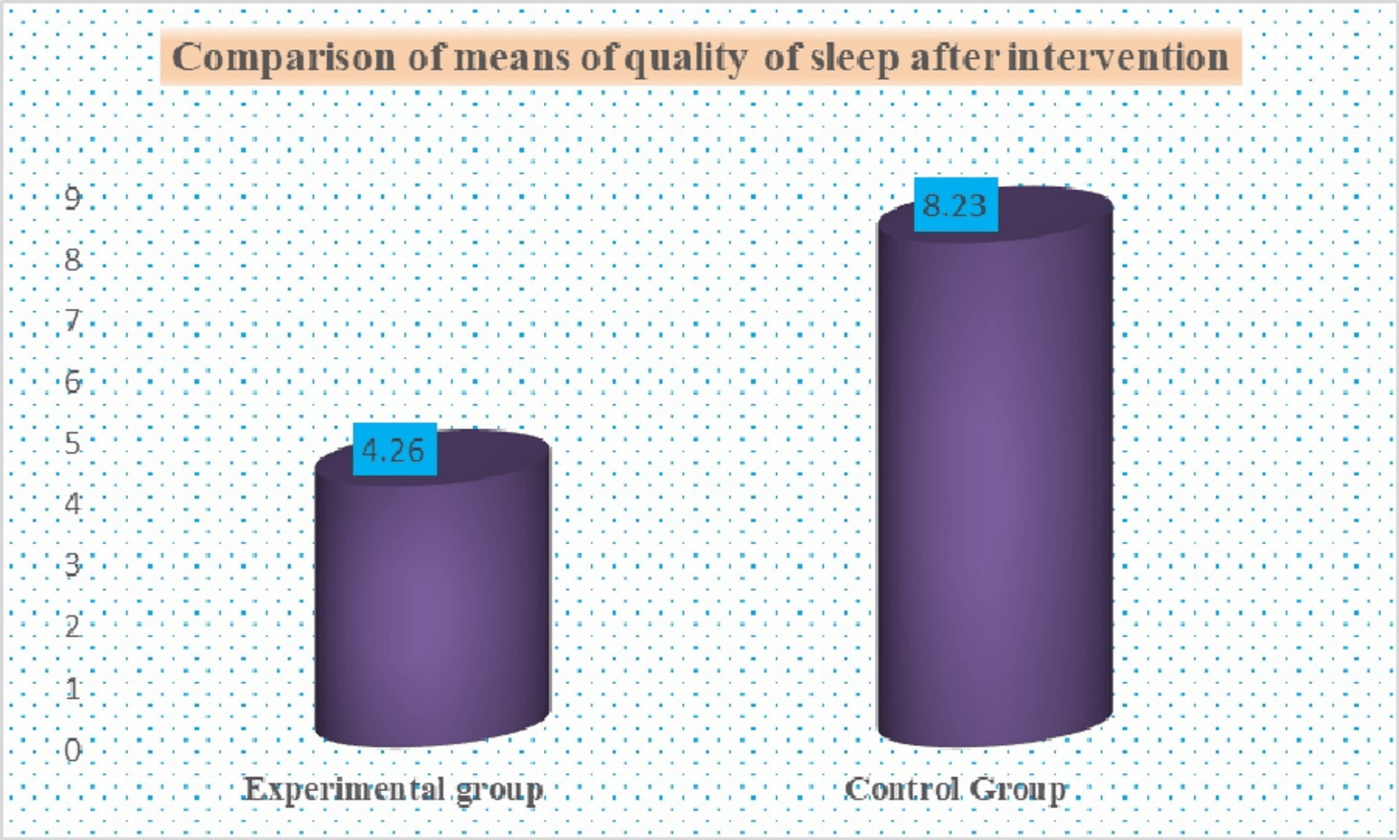
Comparison of the quality of sleep in experimental and control group after intervention

According to the PSQI score, the quality of sleep among old-age individuals in the experimental group mean was 4.26, S.D. was 1.7207, and in the control group mean was 8.23, S.D. was 2.4166, and t-value was 7.3234, and p-value was 0.00001 < 0.05 (at 5% level of significance). The comparison between the experimental and control groups of sleep quality after the intervention is presented in Figure 5.

**Figure 6:**
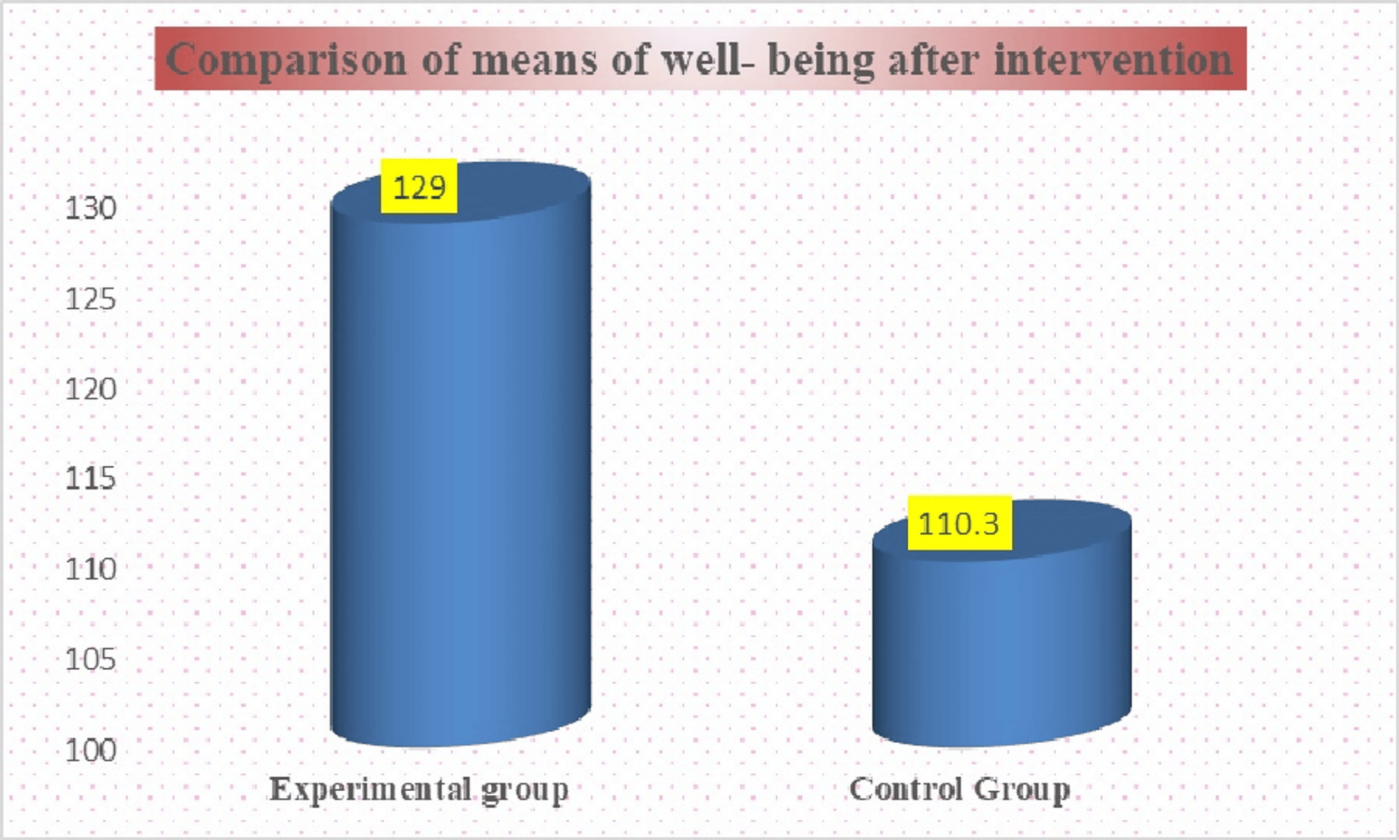
Comparison of well-being in experimental and control groups after intervention

According to the well-being score among old-age individuals in the experimental group, the mean was 129, S.D. was 13.3209, and in the control group, the mean was 110.3, S.D. was 8.065, and the t-value was -6.5773, and the p-value was 0.00001 < 0.05 (at a 5% level of significance). The comparison between the experimental and control groups of well-being after the intervention is presented in Figure 6.

## Discussion

The findings of the study indicate a significant improvement in sleep quality and overall well-being among older adults following the implementation of a calisthenics exercise program. Calisthenics exercises are a form of physical activity that involves using your body weight as resistance to improve strength, flexibility, balance, and overall fitness. These exercises are typically performed without the use of specialized equipment, making them accessible and versatile. They focus on functional movements that engage multiple muscle groups simultaneously and can be easily adapted to suit various fitness levels, including elderly individuals. The pre-test and post-test scores revealed marked progress in the participants' outcomes, supporting the acceptance of hypothesis H1. These findings align with existing literature, which highlights the positive effects of physical exercise on geriatric health.

King et al. demonstrated that moderate-intensity exercises, such as calisthenics, significantly improve sleep efficiency and reduce sleep latency among elderly individuals [10]. Reid et al. concluded that structured physical activity enhances physical and mental well-being, including reduced symptoms of depression and anxiety [11].

Desai et al. review in Cureus delves into the intricate relationship between circadian rhythms, sleep, and recovery. The article highlights the pivotal role of circadian biology in regulating sleep-wake cycles and its impact on physical and mental recovery. By synthesizing current research, the authors emphasize how disruptions in circadian rhythms can impair health and performance, offering insights into optimizing recovery through rhythm alignment. The review is comprehensive and relevant, with practical implications for clinical and lifestyle interventions. While the discussion is thorough, further focus on individualized approaches to circadian rhythm management would strengthen its applicability to diverse populations [12].

Purwanto's study in the Formosa Journal of Science and Technology explores the correlation between physical activity and sleep quality in the elderly. It underscores the positive impact of regular physical activity on improving sleep patterns among older adults. The findings are based on robust data, highlighting physical activity as a non-pharmacological intervention for enhancing sleep quality. This article contributes significantly to geriatric care by emphasizing lifestyle modifications to address common sleep disturbances in aging populations. While insightful, the study could benefit from more diverse sample populations to enhance generalizability. It is a valuable resource for researchers and healthcare professionals [13].

The existing literature provides a substantial basis for the investigation of how physical exercise, particularly calisthenics, can positively impact the quality of sleep and overall well-being in older adults. As mentioned in the study, it is essential to consider the findings from studies similar to yours, especially those conducted in institutional settings like old age homes or nursing facilities. This study underscores the role of calisthenics exercises as a cost-effective and accessible intervention to enhance the quality of life in the elderly. By bridging the gap between physical activity and geriatric health outcomes, these findings support the integration of structured exercise programs into routine care for older adults. Future research may focus on exploring long-term benefits and potential adaptations for individuals with limited mobility.

We acknowledge some limitations of the study. The findings are limited by the small sample size of 60 elderly individuals, which may restrict the generalizability of the results. The data collection period of two weeks may have limited the depth of the information gathered. Additionally, the study was conducted only in selected old age homes within the Sangli, Miraj, and Kupwad corporation areas, which may not represent the experiences of elderly individuals in other regions.

## Conclusions

The study concludes that calisthenics exercises are an effective intervention for improving the quality of sleep and well-being among elderly individuals. The results highlight a correlation between the pre-test and post-test scores and demographic variables such as age, gender, regular exercise habits, and the duration of staying in old age homes. It was observed that a significant proportion of the elderly population suffered from poor sleep quality, which negatively affected their overall well-being. The introduction of calisthenics exercises led to a notable improvement in both sleep quality and general well-being, as evidenced by the marked difference between pre-test and post-test scores. These exercises, being simple and adaptable, provided an accessible means to enhance physical and mental health in older adults. The findings underscore the importance of integrating physical activity programs into the routine care of elderly individuals, particularly those residing in old age homes, where opportunities for physical activity may be limited. This study provides valuable insights that can be utilized in various healthcare settings to promote geriatric health. It emphasizes the need for structured and sustainable exercise programs tailored to the needs and capabilities of older adults. Moreover, the results suggest potential for further development and refinement of such programs to maximize their benefits. Future research may focus on exploring long-term effects, personalized exercise regimens, and strategies to increase adherence, ensuring that the positive impacts on sleep quality and well-being are sustained over time.
